# Aptamers and Nanobodies as New Bioprobes for SARS-CoV-2 Diagnostic and Therapeutic System Applications

**DOI:** 10.3390/bios14030146

**Published:** 2024-03-15

**Authors:** Ki Sung Park, Tae-In Park, Jae Eon Lee, Seo-Yeong Hwang, Anna Choi, Seung Pil Pack

**Affiliations:** Department of Biotechnology and Bioinformatics, Korea University, Sejong 30019, Republic of Korea; pks850528@korea.ac.kr (K.S.P.); jjuft@korea.ac.kr (T.-I.P.); wodjs610@korea.ac.kr (J.E.L.); se003210226@korea.ac.kr (S.-Y.H.); dkssk0714@korea.ac.kr (A.C.)

**Keywords:** SARS-CoV-2, infectious disease diagnosis, biosensing technology, bioprobe, aptamer, nanobody

## Abstract

The global challenges posed by the severe acute respiratory syndrome coronavirus 2 (SARS-CoV-2) pandemic have underscored the critical importance of innovative and efficient control systems for addressing future pandemics. The most effective way to control the pandemic is to rapidly suppress the spread of the virus through early detection using a rapid, accurate, and easy-to-use diagnostic platform. In biosensors that use bioprobes, the binding affinity of molecular recognition elements (MREs) is the primary factor determining the dynamic range of the sensing platform. Furthermore, the sensitivity relies mainly on bioprobe quality with sufficient functionality. This comprehensive review investigates aptamers and nanobodies recently developed as advanced MREs for SARS-CoV-2 diagnostic and therapeutic applications. These bioprobes might be integrated into organic bioelectronic materials and devices, with promising enhanced sensitivity and specificity. This review offers valuable insights into advancing biosensing technologies for infectious disease diagnosis and treatment using aptamers and nanobodies as new bioprobes.

## 1. Introduction

Coronavirus disease 2019 (COVID-19), caused by severe acute respiratory syndrome coronavirus 2 (SARS-CoV-2), first appeared in bats in China, spreading to the species thought to be an intermediate host and then to humans [1]. Despite the widespread presence of coronaviruses in animals, seven pathogenic coronaviruses have been identified in humans over the last 20 years [1], with three of them raising special concern due to high pathogenicity, causing severe pneumonia and death [2]. SARS-CoV, HCoV-NL63, HCoV-229E, HKU1, HCoV-OC43, and Middle East Respiratory Syndrome Coronavirus (MERS-CoV) are examples of human coronaviruses (HCoVs). While HCoV-NL63 and HKU1 were initially reported in 2004 and 2005, respectively [3,4], HCoV-OC43 and HCoV-229E were first discovered in the 1960s [5,6].

SARS-CoV-2 is a member of a broad virus family with the biggest known RNA genome, a single-stranded positive RNA genome containing >30,000 nucleotides [2,7]. The virus comprises several structural proteins, including the nucleocapsid (N), membrane (M), envelope (E), and spike (S) proteins [8,9,10]. Two structural proteins of SARS-CoV-2, S and N proteins, are primary antigens employed in COVID-19 diagnostic tests. The N protein is involved in viral RNA replication, packaging, and transcription, whereas the S protein is involved in the attachment and infection of host cells [11,12]. Unlike other structural proteins, only the S protein is present on the virus surface, allowing rapid detection without viral lysis.

To diagnose SARS-CoV-2 early when it was first discovered, reverse transcription polymerase chain reaction (RT-PCR) has been performed as a gold standard method to detect viral RNA [13,14]. However, RT-PCR requires costly equipment, skilled personnel working in accredited laboratories, and a turnaround time of >24 h to screen and diagnose patients with suspected SARS-CoV-2 infection [15]. To overcome these drawbacks and provide rapid diagnosis, numerous diagnostic approaches have been developed, such as lateral flow-based immunoassays [16] and CRISPR-based assays [17,18]. Although these approaches provide rapid diagnosis, the problems of a high false-positive rate and high production costs remain due to their reliance on antibodies or enzymes [19]. Given these points, numerous research has increasingly focused on investigating the usage of the SARS-CoV-2 S protein as one of the appropriate antigens for creating alternative diagnostic tools to identify viral infection [15,20,21]. Combining the S protein with captivating platforms has recently precipitated a rapid acceleration in the development of advanced diagnostic tools [22].

In advanced biosensors, the binding affinity of molecular recognition elements (MREs) is the pivotal factor dictating the dynamic range of the sensing platform. Moreover, the sensitivity of these biosensors is intricately connected with MRE quality and functional efficacy. This comprehensive review explores the recent advancements of aptamers and nanobodies, emerging as fascinating MREs, particularly in the landscape for SARS-CoV-2 diagnostic and therapeutic applications (Table 1).

Aptamers, often referred to as “chemical antibodies”, are single-stranded oligonucleotides capable of folding into intricate three-dimensional (3D) structures and playing as molecular recognition elements to the specific target [36]. Typically, the length of aptamer is 35–100 nucleotides (nt). Having a ~30 kDa size in the folded state, aptamers act on the target either as ligands for larger receptors or receptors for small molecules [37,38]. Aptamers are capable of binding to extensive targets with high affinity and specificity, ranging from small molecules (metal ions, lipids, and sugar moieties) to macromolecules (proteins and whole cells) [37,39,40,41,42]. Additionally, their relatively small size is advantageous to avoid spatial collisions after binding to the cell or virus surface. Aptamers offer several additional advantages, including notable biostability, facilitated transport and storage, high bioavailability, and low immunogenicity [37,42,43,44,45,46]. Furthermore, aptamers pose conformational switching while preserving their binding abilities [37,47]. In the absence of a target, the aptamers maintain their thermodynamically stable conformation. However, in the presence of the target, the affinity between the two induces binding, forming an aptamer–target complex. Following binding, there is an adaptive trapping of the target by the aptamer, forming stabilized tertiary structures from the secondary structures of the aptamer [37]. Because nucleic acid aptamers enable easy modification and synthesis in large quantities through such chemical methods, utilizing aptamer-based biosensors suggests significant expansion potential in various applications [48].

Another high-performance bioprobe that has recently gained attention is nanobodies, which have several advantages in diagnostic applications. Nanobodies are composed of the variable domain of the heavy chain of heavy-chain-only antibody (VHH), originating from the homodimeric camelid heavy-chain-only antibody (HcAb). They are typically found in members of the Camelidae family (camels, dromedarys, llamas, alpacas, vicuñas, and guanacos) and are analogous to the heavy chain variable (VH) domain found in conventional antibody (Ab) [49,50]. Despite having a monomeric binding region consisting of only three hypervariable loops or complementarity-determining regions (CDRs), nanobodies exhibit high affinities at the nanomolar and sub-nanomolar levels, similar to Ab. Additionally, nanobodies have low immunogenicity, high specificity, and high stability, allowing for rapid generation, large-scale production, and adequate storage and transportation. Nanobodies can recognize more cryptic antigenic epitopes that are difficult to recognize by traditional antibodies, expanding their diagnostic capabilities and exhibiting a strong ability to recognize hidden epitopes [51]. Consequently, these characteristics make nanobodies a viable option for developing advanced diagnostic tools [52].

**Figure 1:**
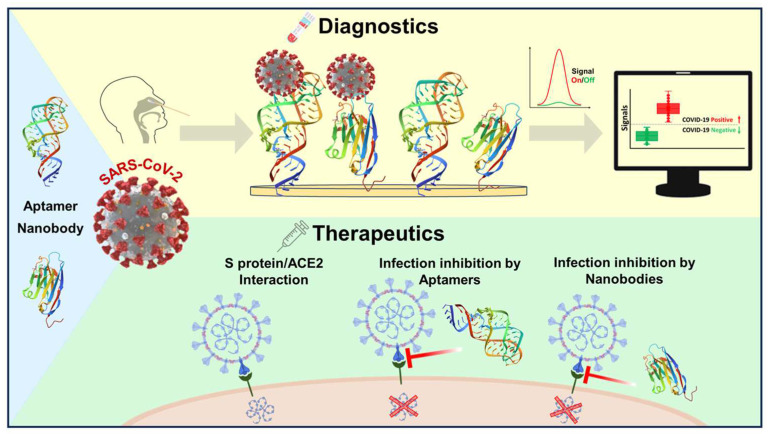
Schematic diagram of the aptamer- and nanobody-based diagnostic and therapeutic applications for SARS-CoV-2.

Aptamers and nanobodies, as advanced MREs, have been widely used in the field of biosensor development. Recently, extensive and sufficient studies have shown their great potential for use in SARS-CoV-2 detection. In this review, we discuss the potential of these advanced MREs to enhance the sensitivity and overall performance of diagnostic and therapeutic tools dedicated to combating the challenges posed by the COVID-19 pandemic (Figure 1).

## 2. Aptamers

With numerous advantages inherent in aptamers, they have become extensively utilized in research fields associated with sensing technology. Aptamers have attracted attention as an essential recognition factor in biosensor design and play a vital role in various analyses and diagnostic applications [48]. Aptamers allow quick and accurate identification of pathogenic organisms in human samples [37,53,54,55]. They act as components of diagnostic analysis to detect specific disease-associated biomolecules and as therapeutic agents by disrupting the function of target molecules. In particular, aptamers represent a practical and inexpensive diagnostic and therapeutic tool for SARS-CoV-2 [42].

### 2.1. Isolation and Characterization of Aptamers through SELEX

**Figure 2:**
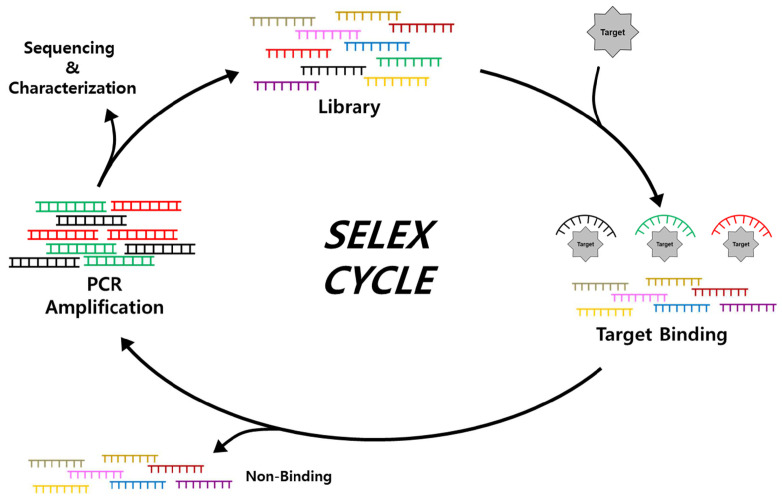
The schematic illustration of systematic evolution of ligands by exponential enrichment (SELEX).

Typically, a novel aptamer sequence that binds to various targets with a high affinity is isolated through an iterative selection process involving target binding, separation, and amplification from the large diversity of a randomized library (Figure 2). Since the systematic evolution of ligands by exponential enrichment (SELEX) was developed by Tuerk and Gold in 1990 [46], discovery research on aptamer use for various targets expanded, with aptamers quickly becoming well-developed innovative materials for numerous applications. Initially, a random library pool contained a random region of up to 60 nt in the center and flanked by a constant region for primer sequences for PCR amplification. Following sufficient binding between aptamer candidates and target molecules, non-binding sequences are removed by various separation strategies depending on the type of SELEX approach. Separation efficiency determines the number of selection rounds in the screening process. Efficiently separated aptamer candidate sequences are then amplified through PCR to obtain an adequate amount for the next process. Single-strand generation is performed by several strategies to generate an enriched pool for the next round of screening [56]. After multiple SELEX cycles, the converged sequences are identified by sequencing analysis [57,58], and their affinities and binding specificities are measured.

Advanced SELEX methodologies have been developed [59], such as capillary electrophoresis-based SELEX [60], magnetic bead-based SELEX [61], and FACS-based SELEX, to improve the efficiency of isolating high-affinity aptamers based on the before-mentioned iterative processes [62]. Furthermore, cell-based SELEX [63] and virus-based SELEX [64] have been developed to enhance target specificity and sensitivity in various environments. High-affinity, high-specificity aptamers obtained by these SELEX methods have several advantages over Abs (Table 1) [23,25,33,56,65,66]. In vitro selection-based screening allows for a shorter selection to produce high-affinity MREs. Additionally, they exhibit high sensitivity even to small target molecules [67,68,69,70,71] and have a relatively compact size, low molecular weight, and high production efficiency. Furthermore, nucleic acid aptamers have higher thermal and pH stability than proteins, enabling conformational changes with reversible denaturation. Owing to these features, nucleic aptamers have attained interest as diagnostic and therapeutic tools [66,72,73,74].

### 2.2. Applications of Aptamers for SARS-CoV-2 Diagnostics

Aptamers have many advantages and higher potential as a diagnostic tool than antibody- or enzyme-based approaches [75,76]. This session introduces aptamer-based biosensors that employ various advanced strategies, including optical, electrochemical, and nanomaterial-combined sensing (Table 2).

#### 2.2.1. Optical Sensing

Several advanced aptasensors have been developed to improve early diagnosis of SARS-CoV-2 with rapid, accurate, and user-friendly diagnostic tools. These aptasensors utilize optical signals and employ various strategies, including colorimetric, fluorometric, and chemiluminescence methods, to discriminate and quantify targets. High sensitivity and specificity in complex environments are crucial for detecting low levels of biomarkers or pathogens. Achieving this requires collaboration between different advanced materials, often involving nanomaterials [113]. Nanomaterials are substances with a size of 1–100 nm, and each material or form has unique chemical, physical, and biological properties [114]. Consequently, aptasensors incorporating nanomaterials demonstrate enhanced performance in electrochemical sensors by leveraging the distinctive properties of various materials in conjunction with aptamers. Moreover, advanced technologies such as surface plasmon resonance (SPR), surface-enhanced Raman scattering (SERS), and bio-layer interferometry (BLI) have been extensively employed with aptamers to achieve ultra-sensitivity (Table 2).

**Figure 3:**
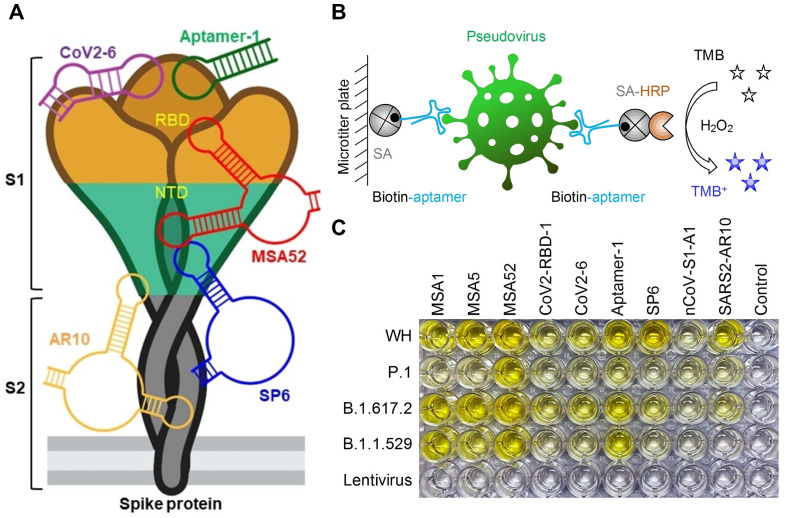
Comprehensive analysis of aptamer performance in SARS-CoV-2 detection. (**A**) The schematic representation illustrates the hypothesized binding sites of various aptamers on the S protein of SARS-CoV-2. RBD, receptor binding domain; NTD, N-terminal domain. (**B**) The schematic illustration of the sandwich assay using pseudovirus. SA, streptavidin; SA-HRP, streptavidin-conjugated horseradish peroxidase. Nine aptamers were utilized in sandwich assays to detect pseudoviruses expressing S proteins from the SARS-CoV-2 wild-type (WT), P.1, B.1.617.2, and B.1.1.529 variants. (**C**) The data show color changes by detection outcomes for pseudoviruses using various aptamers. Reprinted from [117].

Regarding the colorimetric assay, it is worth noting that it is the simplest, most basic, highly visible, and relatively inexpensive method available for detection [79,83,88,115,116]. A representative example is the enzyme-linked aptamer-based sandwich assay (ELASA), which utilizes aptamers instead of Abs in an enzyme-linked immunosorbent assay (ELISA). The primary aptamer, as a capture probe, is conjugated with a specific material surface using biotin–streptavidin interaction or gold–thiol conjugation, and the horseradish peroxidase (HRP)-conjugated secondary aptamer is used as a detection probe. With SARS-CoV-2 captured by the primary aptamer, the secondary aptamer forms a sandwich-type complex, amplifying the signals through catalytic oxidation of 3,3′,5,5′-tetramethylbenzidine (TMB) by HRP. Utilizing this ELASA, Li et al. [79] achieved sensitive detection of pseudo-lentiviruses with a limit of detection (LOD) of 400 fM in 50% saliva. Furthermore, Zhang et al. [117] utilized nine previously discovered SARS-CoV-2 aptamer sequences to detect several variants of the S protein of pseudoviruses and determine the performance of these aptamers for variants of concern (VOCs) recognition (Figure 3). Other colorimetric strategies used absorbance and color change methods. For example, inactivated SARS-CoV-2 can be detected by the wavelength shift in surface plasmon absorbance caused by the aggregation of aptamer-functionalized gold nanoparticles (AuNPs), achieving an LOD of 16 nM for the S protein and high sensitivity for inactivated SARS-CoV-2 of 3540 genome copies/μL. [83]. The strategy of rolling circle amplification (RCA)-urease based biosensing has also been reported for the detection of SARS-CoV-2 [88]. This sensor exploits the yellow-to-pink transition of phenol due to urease activity enhanced by RCA, achieving an LOD of 3.2 × 10^3^ copies/mL for pseudovirus.

In a sensor, response speed and sensitivity are important, as well as ensuring good accessibility to enable point-of-care (POC) testing. Colorimetric sensors are widely used due to their affordability and simplicity. Aloraij et al. [95] reported the development of a colorimetric assay for SARS-CoV-2 variants that can be read out visually by non-skilled personnel. The carboxy-functionalized nanospheres were used as a visualization tool to display different colors (blue, yellow, red, and orange) depending on the target, allowing variants to be distinguished within 4 min with an LOD of 100 ng/mL. The lateral flow assay (LFA) is also the most common colorimetric sensor. LFA employs a sandwich assay, in which SARS-CoV-2 is attached to the bottom-anchored aptamer, and another aptamer modified with AuNPs or enzymes is additionally bound to SARS-CoV-2. Subsequently, AuNPs and enzymes can be used to visualize the presence of SARS-CoV-2 [96,97,99]. Multiplexed LFA can obtain and detect targets from various samples, as well as identify multiple targets at the same time [98].

**Figure 4:**
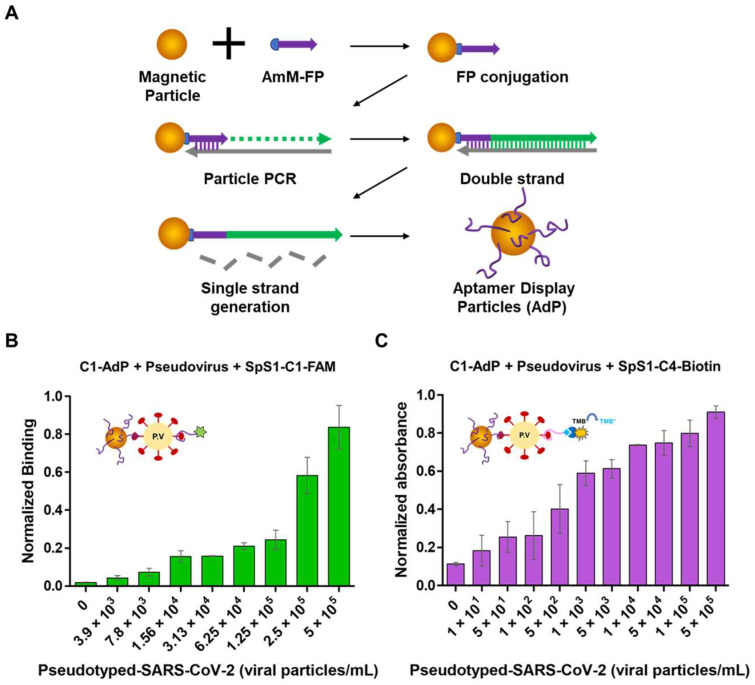
The AdP generation process and schematic. (**A**) The process of producing AdP. FP beads are created by conjugating the AmM-FP on the magnetic beads using EDC/NHS chemistry. Particle PCR is used on the FP bead to amplify the unique aptamer sequence. Alkaline denaturation creates single-strand generation after PCR amplification to remove the antisense strand. (**B**) SpS1-AdPs-based sandwich-type fluorometric SARS-CoV-2 pseudovirus assay. (**C**) SpS1-AdPs-based sandwich-type colorimetric SARS-CoV-2 pseudovirus assay. To identify different quantities of pseudoviral particles, the sandwich-type colorimetric assay utilized C1-AdP as the primary capture probe and biotin-modified SpS1-C4 aptamer as the secondary detection probe. Avidin and HRP were added for signal amplification after the sandwich formed. Reprinted from [81].

With the aforementioned features of nucleic acid aptamers, it becomes possible to achieve sensitive detection of SARS-CoV-2 even without advanced sensing technologies. Park et al. reported the aptamer display particles (AdPs) generated with high-quality aptamers that allow easy fabricating of the SARS-CoV-2 detection matrix by particle PCR, resulting in sufficiently reliable signals and diagnostic accuracy without advanced platforms. Using SARS-CoV-2 S protein-binding aptamer displayed particles, referred to as SpS1-AdPs, allows precise analysis of individual particles and easily fabricates SARS-CoV-2 diagnostic matrix to be applied to sandwich-type fluorescence and colorimetric analysis (Figure 4) [81]. By evaluating the performance of the AdP-based sandwich-type detection system for the SARS-CoV-2 diagnostic approach using pseudoviruses, fluorescence analysis showed a detection limit of 3.9 × 10^3^ pseudovirus particles/mL, and colorimetric analysis with signal amplification achieved high sensitivity with a detection limit of 1 × 10^1^ pseudovirus particles/mL. This demonstrates a competitive sensitivity even without an advanced detection platform for SARS-CoV-2 detection.

The fluorometric approach is a commonly used method for visualizing viral infection and is more sensitive and accurate than most colorimetric systems [115]. Using a fluorophore–quencher pair is the primary method of fluorometric assay. The fluorescence is not observed in the absence of target molecules because the relative distance between the fluorophore and the quencher is close. However, the aptamer undergoes a 3D structural change upon binding to the target, resulting in visible fluorescence caused by moving the fluorophore away from the quencher. Idili et al. [101] used this fluorescence sensor that undergoes a conformational change upon binding to its target, as detected by fluorescence spectroscopy. Additionally, the aptamer/antibody sandwich method for digital detection has been published that using fluorescein-di-β-D-galactopyranoside degradation by β-galactosidase to produce fluorescence in targeting the N protein of SARS-CoV-2. The LOD of N protein in this digital method was 33.28 pg/mL, showing a 300 times lower detection limit than the traditional ELISA method [78]. Fluorescence-linked aptamer assays (FLAA) have been reported as a sandwich-type detection system using fluorescence modification directly on an aptamer without enzymatic linkage [77,104]. As the fluorescence sensor utilized inorganic material, gold nanostar (AuNS), which changed the shape of AuNPs, detected specific SARS-CoV-2 S protein or SARS-CoV-2 pseudo-typed baculovirus in 10 min through distance-dependent nanoparticle surface energy transfer (NSET) spectroscopy (an LOD of 130 fg/mL for antigen and 8 particles/mL for virus) [82]. Furthermore, methods incorporating aptamers into microfluidics (an LOD of 37 active virions/μL for S and E proteins) [89] or nanoporous anodic alumina (an LOD of 7.5 × 10^3^ plaque-forming units (PFUs)/mL for S protein) [100] have been developed to monitor community safety and the environment, as well as to test and track infected individuals quickly and accurately.

Chemiluminescence (CL)-based detection is a highly sensitive and selective approach with several advantages. CL is simple and easy to operate without an external light source and has a higher signal-to-noise ratio than the fluorescence method. Therefore, CL has been widely used in various optical sensing applications [118]. Kacherovsky et al. [80] discovered a high-affinity aptamer SNAP 1 that binds to the N-terminus domain (NTD) of the SARS-CoV-2 S protein. The authors performed aptamer/antibody sandwich ELISA for ultraviolet (UV)-inactivated SARS-CoV-2 virus detection via luminescence reaction of luminol, resulting in sensitive detection at concentrations as low as 5 × 10^5^ copies/mL.

In addition to labeling approaches with enzymes, fluorescent dyes, and CL in optical biosensors, label-free approaches on the probe have been established and combined with susceptible platforms, such as SPR, SERS, and BLI technologies. For example, a label-free SPR-based aptasensor was reported, in which the interactions between aptamer and SARS-CoV-2 S protein receptor-binding domain (RBD) were sensitively monitored. In this aptasensor, the RBD aptamer was immobilized on the SPR D-shaped plastic optical fiber (POF) probe and its binding to the target was optically sensed without any amplification system, resulting in an LOD of 36.7 nM for RBD in the S protein [92]. Zhang et al. characterized the aptamer sequences for the N protein of SARS-CoV-2 through SPR and evaluated the potential of sandwich-type applications using a combination of aptamers [96]. The N protein-binding aptamer was immobilized on niobium carbide MXene quantum dots to enhance the SPR response to achieve ultra-sensitivity while using the SPR aptasensor. This strategy demonstrated the method of N-gene aptasensor fabrication and a new sensing strategy for the sensitive analysis of SARS-CoV-2 N-gene [93]. Localized SPR (LSPR) using nanoparticles was employed to also achieve ultra-sensitivity while using the SPR aptasensor. LSPR-based aptasensors had much higher sensitivities with an LOD of 0.26 nM for the S protein subunit 1 (S1) [84] and an LOD of 9.2 pM for the N protein [85].

**Figure 5:**
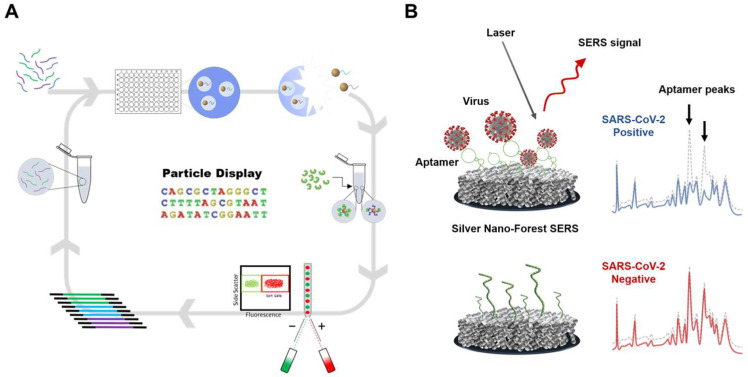
Label-free SERS-based aptasensor using high-throughput screened DNA aptamers. (**A**) Schematic illustration of the particle display aptamer discovery process. (**B**) Aptamer conjugated to a silver nanoforest (SNF) substrate for the detection of SARS-CoV-2. The intrinsic peak of the aptamer shifts in response to conformational changes induced by S protein binding. Reprinted with permission from [73]. Copyright © 2023 Elsevier.

In the last decade, nucleic acid aptamer-based SERS biosensors have been intensively established for the ultra-sensitive detection of various analytes. The drawback that was not inherently ideal for biological applications of Raman scattering was overcome by providing up to 10^8^-fold enhancement of the Raman signal [73]. The spherical cocktail aptamers-gold nanoparticles (SCAPs) as a SERS substrate and nanoprobes with the Raman reporter have been employed to achieve ultrasensitive detection. When substrate and nanoprobe form a sandwich structure with the SARS-CoV-2 S protein, the SERS signal generates ultra-strong “hot spots”, enabling a highly sensitive detection of SARS-CoV-2. This system achieved an LOD lower than 0.7 fg/mL for the S protein and 0.8 transduction unit/mL for the SARS-CoV-2 pseudovirus [87]. Recently, ultra-sensitive detection of SARS-CoV-2 variants using a label-free SERS-based aptasensor has been successfully achieved even without a Raman reporter. Two DNA aptamers were isolated by particle display that exhibited Kd values <2 nM. These aptamers were conjugated on a silver nanoforest (SNF) substrate, enabling observation of the intrinsic aptamer signals. This intrinsic signal-based approach works without Raman labeling on the probe. This approach is based on the fact that the aptamer structure is switchable into 3D folding upon binding with the target molecules. Using this ultra-sensitive label-free SERS-based aptasensor, the attomolar (10^−18^ M) level of LOD was achieved for the S protein, and excellent accuracy for universal detection of SARS-CoV-2 variants was proved in clinical samples (Figure 5) [73].

BLI, which analyzes the interference patterns of white light reflected from the surface of BLI sensor tip, has recently received increased attention over the SPR-based approach. BLI is a technique for detecting targets by immobilizing probes or analytes on the end of a transparent columnar tip and calculating the difference in wavelength arrival times upon further binding interactions. Poolsup et al. reported the BLI-based label-free aptasensor for SARS-CoV-2 N protein detection. This label-free aptamer-based BLI detected the SARS-CoV-2 N protein of the wild-type (WT) virus, as well as Delta and Omicron variants. This approach had an LOD of 4.5 nM for the N protein and successfully detected it in human saliva [91]. Another reported technique included a nanointerferometer sensor with aptamers as recognition elements, in which SARS-CoV-2 RBD-binding aptamer was incorporated into a nanostructured Fabry–Perot interference (nanoFPI) platform. The authors explored the rapid optical detection of mutated RBD from SARS-CoV-2 VOCs and showed that the presence of pseudovirus_WT_, pseudovirus_beta_, and pseudovirus_gamma_ could be detected with significant optical signal shifts at as low as 4 TCID_50_/mL [90].

#### 2.2.2. Electrochemical Sensing

**Figure 6:**
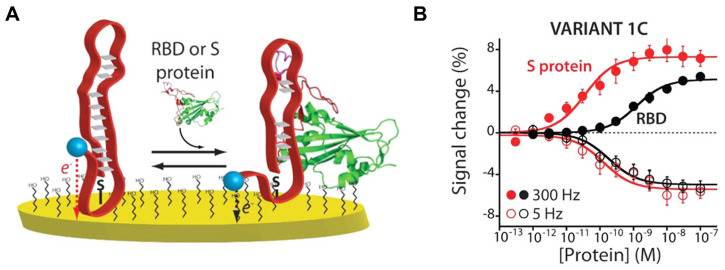
The electrochemical aptamer-based (EAB) sensor. (**A**) The aptamer was modified with an Atto MB2 redox reporter and attached via a hexanethiol anchor to a gold wire electrode coated with a self-assembled monolayer of 6-mercapto-1-hexanol to support the electrochemical readout. (**B**) The EAB sensor responds to the increasing concentrations of the SARS-CoV-2 RBD (black curves) and the S protein (red curves), producing the expected Langmuir binding curves. The signal-on response of EAB sensor was displayed at higher square wave frequencies (300 Hz), and a signal-off response was displayed at lower frequencies (5 Hz). Reprinted from [101].

An electrochemical sensor detects chemical changes around the device and converts the signal into electric current or electrical resistance on the electrode surface. Electrochemical sensors can be used repeatedly with high sensitivity [112,119,120]. For example, Idili et al. developed an electrochemical aptamer-based (EAB) sensor [101] for the SARS-CoV-2 S protein to detect changes in the current occurring under aptamer-target binding. Specifically, the ends of surface-immobilized aptamers were modified with redox reporters called methylene blue derivatives, which exchange electrons with the interrogating electrode upon binding to their target, placing them further or closer to the electrode surface (Figure 6). Martinez-Roque et al. also reported the EAB sensor [104], which utilizes the same method, achieving an LOD of 8.85 fg/mL for the S protein. In addition, the aptamer was immobilized on AuNPs on the carbon electrode to improve the detection time of SARS-CoV-2, and the electrochemical impedance was analyzed after incubation for 40 min at various target concentrations, resulting in an LOD of 66 pg/mL for SARS-CoV-2 RBD [107]. Furthermore, Han et al. [108] reported aptamer dimerization inspired by the biomimetic clamp assay for SARS-CoV-2 detection, in which a pair of sandwich-type aptamers are dimerized to achieve high-performance target recognition. They incorporated the DNA biomimetic clamp (DNA-BC) into AuNP-decorated amino-functionalized UiO-66 (Au@UiO-66-NH_2_)-modified screen-printed gold electrodes (SPGEs) to form adsorption sites and enhance the electrical conductivity. Using this integrated DNA-BC/Au@UiO-66-NH_2_/SPGE impedimetric aptasensor, ultrasensitive detection was achieved with a detection limit as low as 0.31 pg/mL for the SARS-CoV-2 N protein.

Moreover, a technology to increase detection sensitivity by amplifying the signal through a hybridization chain reaction was developed, achieving an LOD of 9.79 fg/mL [103] and 0.75 fM [102] for the S protein. Various types of ultrasensitive electrochemical sensors have also been developed using the unique ion current rectification of nanopipettes (an LOD of 73.204 pg/mL for the N protein) [121] and a solid-state nanopore (an LOD of 1000 copies/mL for SARS-CoV-2) [122].

Additionally, the carbon nanotube-based aptasensor serves as a notable example in this context. Single-wall carbon nanotubes (SWCNTs) can exhibit high electrical conductivity or semiconducting properties and sensitively detect changes in current based on the behavior of surrounding molecules. Using this feature, a folding-based electrochemical aptasensor allows the single-step detection as a target-induced change in current. Curti et al. [106] applied it to developing a rapid and reagent-free electrochemical sensing platform for the single-step detection of the SARS-CoV-2 S protein S1. This platform showed low LOD and limit of quantitation (LOQ) in the low nanomolar range and exhibited high specificity and low cross-reactivity in the presence of interfering viral proteins. In addition, carbon-nanotube-array-based thin-film transistor biosensors (CNT-array TFT), merged with tetrahedral DNA nanostructure (TDN) and aptamer, enhanced sensor responsiveness and protein capture efficiency due to avoiding the aggregation or entanglement of aptamers, achieving an LOD of 10 aM (6 copies/μL) for SARS-CoV-2 WT RBD and 6 aM (3.6 copies/μL) for Omicron RBD [109]. Furthermore, a sensor using quantum dots with optical and electrical properties as fluorophore reporters [105] and a sensor using a biomimetic nanochannel with rapid and sensitive performance for biomolecules were also constructed [110], achieving an LOD of 0.5 pg/mL for the S protein and an LOD of 1 fM for the S protein S1, respectively.

**Figure 7:**
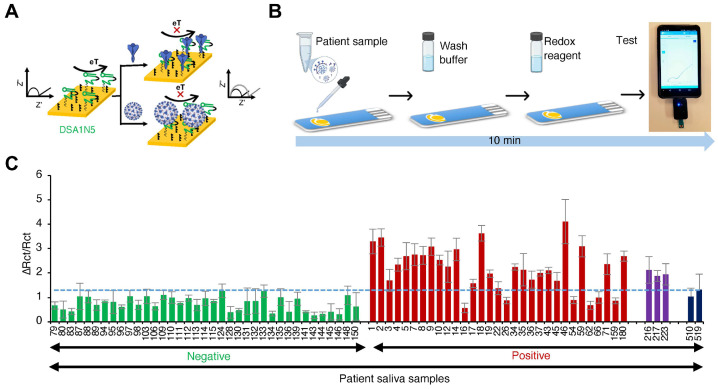
Collaboration of the portable device and rapid electrochemical sensor for detecting WT and B.1.1.7 SARS-CoV-2 variants in unprocessed saliva. (**A**) The schematic of the electrochemical assay for detecting SARS-CoV-2 using an S protein-binding aptamer. (**B**) Illustration of the detection process. Following dilution, the sample was added to the electrode, incubated for 5 min at room temperature, washed for 1 min, and scanned for 2 min using a handheld mobile-operated potentiostat. (**C**) The graph shows the distribution of negative patient saliva samples (green bar) and COVID-19 positive patient saliva samples (red, purple, and black bars represent the original virus, Alpha, and Delta variants, respectively). Reprinted from [112].

Although electrochemical sensors are limited to POC use due to the need for accurate measurement equipment, they have recently been combined with portable devices that are relatively less sensitive but can be used as diagnostic tools. For example, Zhang et al. [112] reported that SARS-CoV-2 S protein-specific dimeric DNA aptamer was immobilized onto gold electrodes to produce an electrochemical impedance sensor, which measures potentiostat by connecting a handheld mobile to an EAB sensor using a gold electrode chip (Figure 7). Using this portable platform, the authors evaluated 36 positive and 37 negative patient saliva samples, resulting in clinical sensitivity of 80.5% and specificity of 100%. Furthermore, the sensor could detect the WT virus, as well as the Alpha and Delta variants, in patient samples.

Another POC electrochemical sensor indirectly detects SARS-CoV-2 using a glucometer [111]. This sensor was initiated by dehybridization of invertase-conjugated antisense strand by aptamer-target binding. Then, the released antisense-invertase converts sucrose into glucose, which can be measured using a glucometer. The antisense strand is a sequence eluted by the binding of an aptamer with its target. Additionally, numerous glucometers could connect to smartphones, allowing for integration with contact tracing apps, medical providers, and electronic health records.

#### 2.2.3. DNA Nanostructures with Aptamers

DNA, the molecule that stores and transmits genetic information in biological systems, is a central building block in DNA nanotechnology, which takes this molecule out of its biological context and uses it to construct structural motifs. The impact of DNA nanotechnology on nanoscience and nanotechnology was revolutionized by the ability to control molecular self-assembly [123]. Unlike other exogenous materials, DNA is almost non-toxic and highly biocompatible. It also has more benefits, including predictability with base-pairing and the ability for programmable and controllable self-assembly. These make DNA a promising building block for constructing DNA nanomaterials with predesigned structures. These are easily modified with nucleic acid probes or directly designed as sensing probes, providing novel insights and opportunities to advance biological applications using nucleic acid probes [124]. In recent years, integrating aptamers and DNA nanotechnology has significantly progressed in diagnostics and therapeutics applications. The convergence of nucleic acid aptamers and DNA nanotechnology has given rise to powerful nanoplatforms used in biosensing, bioimaging, targeted drug delivery, bioregulation, and biomimicry [125]. In this chapter, we discuss the response to SARS-CoV-2 by combining DNA nanostructure with aptamer as an advanced biosensing platform.

**Figure 8:**
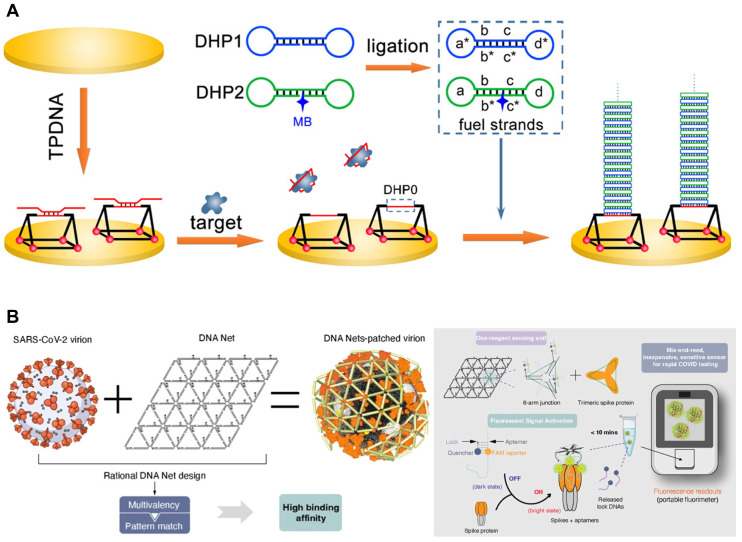
The scheme of strategies based on DNA nanostructure as scaffolds in combination with aptamer. (**A**) Schematic representation of the TPDNA assembly process, multi-thiol-assisted electrode modification, aptamer-mediated recognition of target proteins, and subsequent DHCR process atop TPDNA. (**B**) Designed DNA Net use aptamers to form trimeric clusters, matching the spacing of protomers on the SARS-CoV-2 S protein. The DNA Net sensor employs FAM-tagged aptamers quenched by partially complementary “Lock” DNA. Virus interaction triggers rapid Lock DNA release, allowing the unquenching of FAM reporters at low viral concentrations for detection sensitivity using a portable fluorimeter. Reprinted with permission from [94,102]. Copyright © 2022 American Chemical Society.

Triangular prism DNA (TPDNA) is a 3D DNA structure that can serve as a template for the growth of new materials, act as a vehicle for drug delivery, and function as an excellent scaffold [102,126]. The TPDNA nanostructure also provides an excellent scaffold, enhancing molecular recognition efficiency on the top single-strand region of TPDNA (DHP0). A study applied DHP0 as the trigger of a dumbbell hybridization chain reaction (DHCR) for electrochemical biosensing (Figure 8A). The SARS-CoV-2 S protein S1 binding aptamer sequence was initially localized through partial hybridization with DHP0. The aptamer sequence was displaced in the presence of SARS-CoV-2 S protein, exposing DHP0. The subsequent introduction of fuel strands in DHCR provided a significantly amplified electrochemical response by initiating compressed DNA linear assembly. The resulting product could be stacked on the TPDNA nanostructure, facilitating the enrichment of electrochemical species and exhibiting higher signal intensity than traditional HCR [102]. Similarly, but with a different structure, TDN has been constructed on a rotational paper-based analytical device (RPAD). The RPAD is simple, portable, and highly efficient, with low production costs. The S protein binding aptamer was utilized on the TDN-constructed RPAD through hybridization between the aptamer and TDN to develop a SARS-CoV-2 diagnostic tool. The pivotal event for SARS-CoV-2 detection was the release from the TDN of the aptamer triggered by the strong binding with the S protein, allowing another biotinylated single-stranded DNA (ssDNA) as a detection probe to replace the aptamer. Afterward, the color changed due to the reaction between HRP and TMB-H_2_O_2_. This strategy showed S protein detection at adequate sensitivity as low as 10–14 g/mL [127].

There are other methods where the target is detected through the interaction with the DNA nanostructure–aptamer complex rather than where the aptamer is released from the DNA nanostructure along with the target for detection. The sensitivity enhancement can be achieved based on the combination within the DNA nanostructure–aptamer complex. One approach involves integrating CNT-array TFT with TDN and triple aptamers to create biosensors. In this method, detection relies on measuring changed current values, where the drain current of CNT-array TFT biosensors undergoes alterations during the sensing process. When positively charged molecules bind to the sensor, the current value increases relative to the same voltage, whereas the current value decreases under the same voltage conditions for negatively charged molecules. Using this principle, the Two-terminal I–V characteristics is measured to detect the presence of the virus when the target binds with the aptamer on TDN [109]. Another approach involves field-effect transistors (FETs) with an electro-enrich able liquid gate (LG) anchored by TDNs. Similar to the aforementioned method, the analyte recognition results in a significant potential change (ΔV_G-E_), which simultaneously induces a remarkable potential change at the graphene channel (ΔV_E-C_) due to the sensitive nature of the graphene surface to charge perturbations [128]. Compared with conventional FETs with probes immobilized on the channel, recognition events at the LG cause a more significant potential perturbation in the channel, providing an amplified electrical response in the channel [128,129,130]. Instead of interacting with detectable proteins in SARS-CoV-2, this method performed the detection by aptamer binding to thrombin. This strategy was based on the consideration that COVID-19 patients exhibit a significant increase in ex vivo thrombin generation by SARS-CoV-2 associated thrombosis [131].

One of the powerful features of the DNA nanostructure–aptamer complex combination is allowing the construction of multivalent aptamers. Multivalent aptamers are molecular constructs that incorporate two or more identical or different aptamers. This multivalency enhances the avidity of aptamers, leading to significantly increased binding avidities compared to monovalent aptamers. Moreover, the multivalency provides aptamer stability, resulting in improved performance under physiological conditions for various applications [132]. For example, using TDN allows for precise spatial organization and matching valence with three neutralizing aptamers. This alignment achieves nanometer-level precision on the topmost surface of the SARS-CoV-2 S trimer, enhancing the interaction with RBD [133] or NTD supersite [134]. As a further design, a net-shaped DNA nanostructure, referred to as the DNA Net, was employed to selectively recognize and capture intact SARS-CoV-2 virions with high affinity. This recognition was achieved through spatial pattern-matching and multivalent interactions between aptamers positioned on the DNA Net and RBD in the trimeric S protein (Figure 8B) [94]. Additionally, an icosahedral DNA framework, termed IDNA-30, was designed to spatially arrange up to 30 aptamers. Each triangular plane of IDNA-30 consists of three precisely positioned aptamers that topologically match the SARS-CoV-2 S trimer, resulting in a multivalent spatially patterned binding [135]. Moreover, employing DNA nanostructures and aptamers, as demonstrated above, has proven as a more effective approach. Recent advancements have extended beyond the scope of treatment or detection, including the development of methods to measure mechanical forces. The mechanical force between a virus and its host cell is pivotal in viral infection. Researchers developed a platform in which the virus is anchored with multivalency-controlled aptamers to facilitate the transfer of the virus-cell mechanical force to a DNA tension gauge tether (virus-TGT). Upon TGT rupture, the binding module–virus–cell complex detaches from the substrate, leading to a reduction in host cell–substrate adhesion. This research revealed the mechanical force between the entire virus and the cell [136].

The amalgamation of DNA nanotechnology and aptamers not only yields innovative results in the fields of ex vivo diagnosis, treatment, and detection but also presents the potential to transform approaches to address various challenges in biomedical applications. The intricate spatial arrangement of DNA nanostructures and aptamer functionality showcase the vast potential for addressing complex issues, such as responding to SARS-CoV-2.

### 2.3. Applications of Aptamers for SARS-CoV-2 Therapeutics

Timely and accurate virus detection is crucial for effectively diagnosing and treating diseases in public health emergencies of international concern. Given the unprecedented spread of SARS-CoV-2 and the severity of the COVID-19 pandemic, there is an urgent need for the enhancement of current diagnostic methods and the accelerated development of respective vaccines and therapeutics [137,138]. Comprehending the entry mechanism of SARS-CoV-2 into human cells is crucial for assessing its infectivity and pathogenesis, as well as devising targeted therapies to counteract it [139]. 

**Figure 9:**
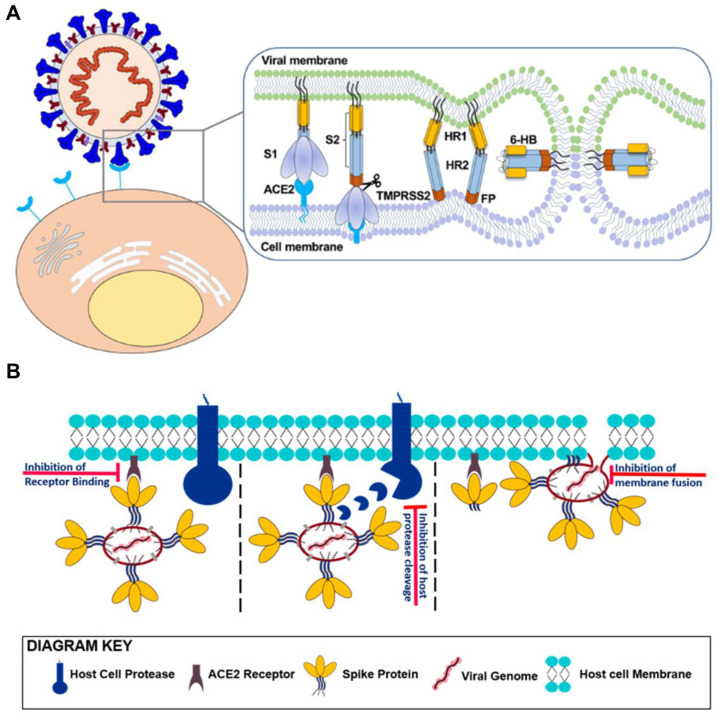
Viral infection mechanism of SARS-CoV-2. (**A**) Entry of SARS-CoV-2 into host cells involves several key steps. The viral S protein initially binds to the angiotensin-converting enzyme 2 (ACE2) receptor, initiating virus attachment to the cell surface. Subsequently, the S protein undergoes cleavage by TMPRSS2, generating S protein subunits 1 and 2 (S1 and S2). Of these, the S2 undergoes conformational changes as the heptad repeats HR1 and HR2 gradually approach each other, forming a six-helix bundle (6-HB). This intricate structural alteration leads to the fusion of the virus envelope with the host cell membrane, allowing viral entry into the host cell. (**B**) The specific site for infection inhibition strategies, including the receptor-binding site, the host protein cleavage site, and the membrane fusion site. The schematic is not drawn to scale; an enlargement has been applied to illustrate the viral entry mechanism more accurately. Reprinted from [139,144].

The initial stage of SARS-CoV-2 entry is contingent upon the interaction between the viral S protein and its cellular receptor angiotensin-converting enzyme 2 (ACE2) [140,141]. After the RBD in the S protein recognizes ACE2, the prefusion trimeric S protein undergoes a series of structural rearrangements through proteolytic cleavage by host cell proteases [139]. This cleavage causes the dissociation between S protein subunits 1 and 2 (S1 and S2) as a post-fusion conformation, in which the fusion peptide in S2 is responsible for membrane fusion between viral and host cell membranes. Once the fusion peptide is inserted into the host cell membrane, trimeric heptad repeats 1 and 2 interact to form a six-helix bundle (6-HB). This leads to viral fusion and RNA genome entry into the host cell [139,141,142,143,144]. Consequently, the SARS-CoV-2 S protein characteristics render it a highly promising target for developing diagnostic and therapeutic methods (Figure 9) [9].

Given the ability to recognize the S protein as described above, nucleic acid aptamers are well-suited for therapeutic applications due to their versatility. Current therapeutics primarily focus on the following aspects: (1) inhibiting the initial attachment and blocking the interaction with ACE2; (2) suppressing the membrane fusion between the virus and host cell; (3) disrupting viral replication, protein processing, and viral assembly/release [145]. Among these mechanisms, inhibiting the interaction between the S protein and human ACE2 (hACE2) stands out as a crucial strategy to prevent viral infection. Research efforts have concentrated on developing aptamers to bind the S protein, and various aptamers have been explored for their potential to inhibit the interaction between the S protein and hACE2 [36]. In this section, we highlight the latest trends in SARS-CoV-2-related aptamers, focusing on aptamers designed to inhibit viral infection in SARS-CoV-2.

#### 2.3.1. Suppressing Initial Attachment: S Protein S1 Targeting Aptamers

Halder et al. developed an aptamer-based system that holds both diagnostic and therapeutic potential for SARS-CoV-2. They isolated several aptamers (R, S, T, U, and W) that efficiently bind with the RBD, with aptamers R and T showing the highest diagnostic potential. Subsequently, they conducted SARS-CoV-2 pseudovirus inhibition assay using these aptamers to identify aptamers with antiviral potential. The antiviral efficiency of the highest binding aptamers R and J (same central sequence as in R but lacking flanked primer regions) showed 95.4% and 82.5% inhibition against SARS-CoV-2, respectively [146].

Sun et al. focused on selecting aptamers with binding sites resembling those of ACE2 against RBD, as they hold promise for blocking viral infection [147,148]. Molecular docking calculations were employed to fasten the aptamer selection process, systemically model minimized energy structures of the aptamer–target complex and predict potential binding sites. The CoV2-6 aptamer exhibited the highest ACE2 inhibitory effect among candidate sequences. CoV2-6 was subjected to further modifications and engineering, resulting in a circular bivalent aptamer named CoV2-6C3 (cb-CoV2-6C3), enhancing its stability, affinity, and inhibitory efficacy. The inhibitory ability of the circular aptamer was subsequently validated through a pseudovirus neutralization assay. The SARS-CoV-2 pseudovirus utilized in this assay was packaged with the S protein of SARS-CoV-2 as the surface capsid glycoprotein using a lentivirus packaging system. The findings indicated that cb-CoV2-6C3 exhibited a high affinity for RBD, with a Kd of 0.13 nM, effectively blocking authentic SARS-CoV-2 with a half-maximal inhibitory concentration (IC_50_) of 0.42 nM [147].

Schmitz et al. discovered SP6, an aptamer binding to the SARS-CoV-2 S protein [149]. This study was conducted simultaneously to suppress pseudovirus infection with SP6 and showed a DNA aptamer employing a distinct mode of inhibiting viral infection. Although this aptamer does not engage with RBD, it effectively inhibits viral infection of the SARS-CoV-2 pseudovirus in an ACE2-expressing cell line. This implies that the inhibition of viral infection can occur independently of RBD targeting, suggesting potential efficacy even when the virus is already bound to the cell. These findings open new avenues for suppressing SARS-CoV-2 infection through a previously unexplored mechanism [149].

#### 2.3.2. Membrane Fusion Inhibition Strategies: S Protein S2 Targeting Aptamers

Studies have been reported explicitly concentrating on inhibiting the membrane fusion between viruses and host cells as part of current therapeutics. As mentioned above, the SARS-CoV-2 S protein S2 mediates the fusion of the viral membrane with potential host cells through the heptad repeat region [36]. Therefore, the discovery of anti-S2 aptamers holds significant interest as it might provide novel strategies for treating viral infections. Furthermore, an anti-S2 aptamer can be conjugated with an anti-S1 aptamer to create a fusion aptamer that binds to the S protein at two sites. This approach could potentially enhance the efficacy of blocking the S protein/hACE2 interaction, given that the fusion aptamer presents a more considerable steric hindrance than each aptamer.

Silwal et al. reported that the S protein binding to DNA aptamer labeled MSA52 demonstrated a universally high affinity for the Alpha, Beta, Gamma, Epsilon, Kappa, Delta, and Omicron variants. The MSA52 exhibited high affinities with Kd values ranging from 2 to 10 nM for all S proteins in SARS-CoV-2 variants. Furthermore, it displayed similar binding affinity to variants not originally present in the reselection experiments [150]. In a subsequent study, authors performed the SELEX to discover a DNA aptamer targeting S2 of the WT S protein [36]. The aptamer S2A2C1 possessed the shortest sequence and demonstrated the highest binding affinity with a Kd value of 35 ± 4.3 nM for the S2. The S2A2C1 aptamer has not been shown to bind to the RBD in the SARS-CoV-2 S protein. However, it effectively blocked the interaction between the S protein and hACE2, suggesting an RBD-independent inhibition mechanism. The S2A2C1 aptamer was conjugated with a previously reported anti-RBD aptamer, S1B6C3, to enhance its performance using various linkers to create hetero-bivalent fusion aptamers. An ELISA was conducted to assess how both aptamers inhibited the S protein/hACE2 interaction. The findings revealed that 31% of the S protein interacted with hACE2 in the presence of S2A2C1, but this value decreased significantly to 9% when S1B6C3-A5-S2A2C1 was employed [36].

These findings show that aptamers are versatile in binding to various binding regions of the SARS-CoV-2 S protein, rendering it well-suited as a diagnostic and therapeutic molecular tool to target SARS-CoV-2 and its variants.

## 3. Nanobodies

**Figure 10:**
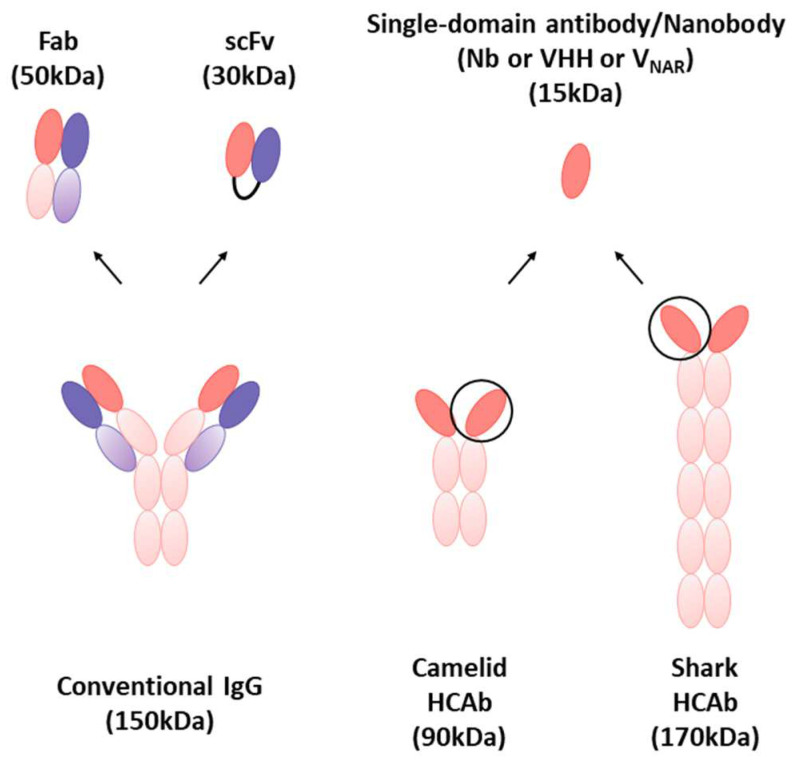
Schematic illustration of conventional antibody and its fragments, heavy-chain-only antibodies, and nanobodies. While conventional antibodies (150 kDa) give rise to fragment antigen-binding (Fab, 50 kDa) and single-chain variable fragment (scFv, 30 kDa), heavy-chain-only antibodies (HcAbs) found in camelids (90 kDa) and sharks (170 kDa) give rise to camelid-derived nanobodies (VHH) and shark-derived nanobodies (VNAR), which are the smallest in size at 15 kDa.

Nanobodies (≈15 kDa) are the smallest functional antibody fragments compared to other existing Abs, such as IgG mAbs (≈150 kDa), HcAb (≈90 kDa), fragment antigen-binding (Fab, ≈50 kDa), and single-chain variable fragment (scFv, ≈30 kDa) (Figure 10) [151]. The size is approximately 2.5 × 4 nm, which is about 10 times smaller than that of a conventional Ab (IgG) (Table 1) [152]. The modularity of nanobodies enables the straightforward creation of multivalent constructs for various applications [27]. Particularly, nanobodies comprise two types of regions with CDRs essential for identifying antigens and the more conserved regions [153]. A nanobody is a fragment of an antibody generated through recombinant technology involving the VHH region amplification [154]. The region with the highest degree of recognition is CDR3 [155], and nanobodies have a longer CDR3 loop, significantly increasing the variety of antigens [156,157]. In monoclonal antibodies (mAbs), CDR1, CDR2, and CDR3 of VH and VL domains contribute equally to antigen binding, whereas CDR3 in nanobodies has a much higher contact ability with the antigen and serves as the primary source of binding affinity [50,158]. Their small size, simple structure, and high antigen-binding affinity make them superior to conventional mAbs [159]. The melting temperature (Tm) of fifteen different types of monoclonal antibodies was evaluated by Kohli et al. using differential scanning fluorimetry (DSF). Every mAb had a Tm of 70 °C or above [160]. Nanobodies also possess remarkable stability in extreme conditions. Mario S Valdés-Tresanco et al. reported that the median Tm for nanobodies from camels, llamas, alpacas, and sharks is 68.5 °C, 68 °C, 66 °C, and 62 °C, respectively. The distribution of all organisms is similar around the mean Tm of 67 °C [161]. Isolated VHH domains are characterized by high solubility, high affinity ranging from nanomolar to picomolar levels, engineering feasibility, tissue penetration, low immunogenicity in humans, and the ability to recognize hidden epitopes [162,163,164]. Owing to these advantages, nanobodies are being researched as potent substitutes for IgG in many biological domains, including diagnosis and therapy.

### 3.1. Isolation and Characterization of Nanobodies through Phage Display

**Figure 11:**
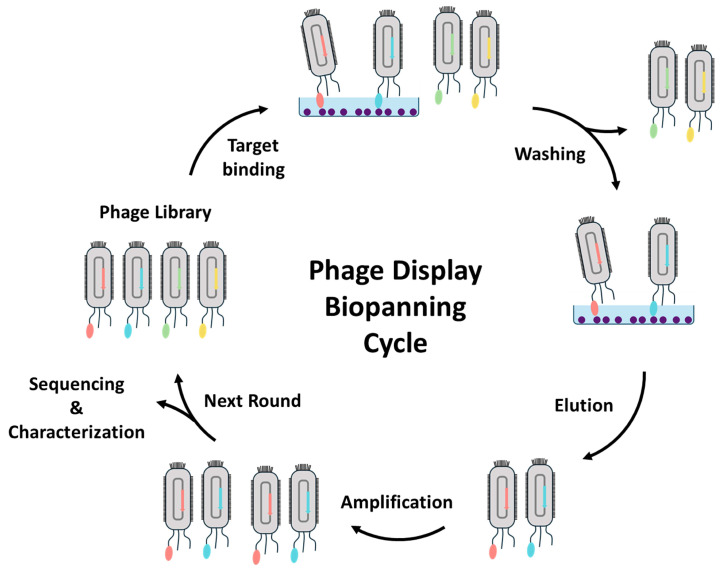
Schematic illustration of phage display biopanning cycle. First, phage display libraries are prepared. Peptides or proteins (such as scFv and nanobodies) can be displayed on the surface of bacterial phages. These displayed molecules can bind to the target. Next, the unbound phages are washed off the target substrate, and the bound phages are eluted from the target substrate. The eluted phages are then amplified in *E. coli*. Subsequently, all steps from binding to amplification are repeated 2–5 times to discover molecules with high affinity.

Phage display technology is the most reliable and widely used method for identifying antigen-specific nanobodies [165]. Phage display was developed by G. Smith in 1985 [166] as a technique for displaying polypeptides on the surface of lysogenic filamentous bacteriophages, enabling the generation of Ab libraries with numerous phage particles [167]. Each phage encodes and displays distinct molecules, resulting in a population of >10^12^ phage molecules, with a diversity ranging from 10^6^ to 10^11^ different ligands [168]. Three categories of nanobody banks can be utilized to obtain antigen-specific nanobodies: immune, naive, and synthetic libraries [27]. The process of screening for antigen-specific nanobodies is referred to as biopanning and is an in vitro selection based on the concept of affinity enrichment. It involves repetitive cycles of incubation, washing, amplification, and reselection of the bound phage based on the binding affinity of clones to select those with desirable traits for a widely used strategy (Figure 11) [168]. The target molecule of interest is normally immobilized on the surface of microtiter plate wells [169], immunotubes [170], PVDF membranes [171], or magnetic beads [172]. Whole cells can also be employed as the target molecule [173]. Through washing steps, phages with low affinity or non-binders are removed, followed by eluting nanobody candidate displayed phages using enzymatic digestion with trypsin or acidic conditions. By utilizing *E. coli* and helper phages, the recovered phages are infected and amplified by bacterial doubling, and then an enriched library is employed in the next round of biopanning [174]. After multiple biopanning cycles, isolated individual candidate clones are characterized using a conventional ELISA to confirm the formation of antigen-specific nanobodies. Subsequently, candidate clones are identified as amino acid sequences by sequencing analysis [175]. Using the identified amino acid sequence of the best nanobody, large-scale nanobody production is allowed by the *E. coli* recombinant system, the most conventional production method.

### 3.2. Applications of Nanobodies for SARS-CoV-2 Diagnostics

With the unique characteristics and advantages of nanobodies compared to conventional mAbs, nanobody-based biosensing applications have recently seen a significant rise in the diagnostic field [159]. Nanobodies, derived from the VHH domain of camelid Abs, have broadened the range of molecules utilized in antigen detection. Their notable attributes, including high affinity for target antigens, compact structure, exceptional stability, and straightforward production, are responsible for their utility as diagnostic reagents [176]. In this session, we introduce a nanobody-based sensor for detecting SARS-CoV-2, including optical sensing, electrochemical sensing, and DNA nanostructure combined applications (Table 3).

#### 3.2.1. Optical Sensing

**Figure 12:**
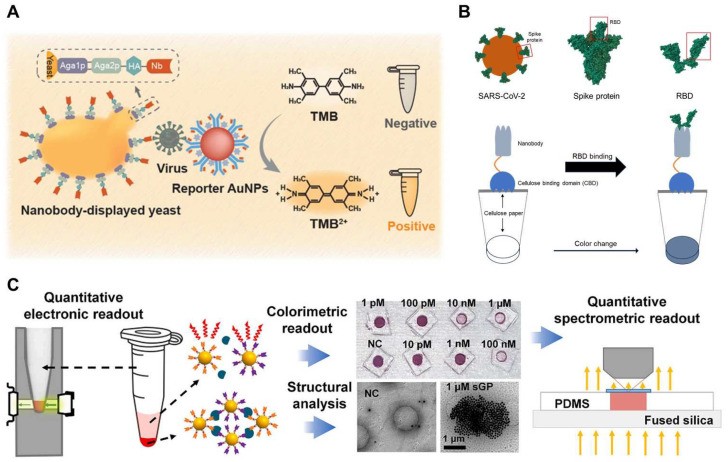
Optical analysis for detecting SARS-CoV-2 based on labels using nanobody. (**A**) The scheme of the nanobody-displayed whole-cell biosensor designed for the colorimetric detection of SARS-CoV-2. (**B**) Schematic of an immunoassay to evaluate fusion protein function by immobilizing nanobody-CBD fusion proteins on cellulose paper and submerging them in RBD-Fc-containing medium. Confirmation of capture capability was performed using anti-human Fc-HRP and 3,3′-Diaminobenzidine Tetrahydrochloride (DAB) substrate. (**C**) Schematic illustration of nanobody-functionalized nanoparticles for rapid electronic detection (Nano2RED) assay. The unique properties of AuNPs with color changes based on the LSPR were employed to eliminate the requirement for signal labeling. AuNP aggregation and sedimentation assay mechanism was characterized with colorimetric readout and structural analysis for quantitative assessment of antigen concentration. Reprinted with permission from [177,181]. Copyright © 2023 American Chemical Society and Copyright © 2022 Elsevier.

ELISA is one of the most commonly used for optical sensing platforms. Sandwich ELISA is frequently employed due to its high specificity and accuracy. Nanobodies are widely utilized in ELISA, specifically in sandwich ELISA using diverse combinations of engineered nanobodies to detect the S [176] and N [180] proteins of SARS-CoV-2. Since ELISA uses one ligand to capture and the other to detect the target analyte, researchers investigated various combinations of engineered nanobodies to replace mAb in sandwich ELISAs and detect SARS-CoV-2 in the fluid. They also identified an optimal combination of nanobodies. Additionally, the results showed that the performance was similar to that of mAb sandwich ELISA. As the other example, a whole-cell biosensor displaying nanobodies were developed for colorimetric detection of SARS-CoV-2 S proteins. Yeast surfaces acting as bioreceptors were genetically engineered to display nanobodies binding to SARS-CoV-2 with high efficiency. HRP-functionalized anti-SARS-CoV-2 S protein S1 mAb was conjugated with AuNPs as signal transducers to achieve sensitive detection by signal amplification (Figure 12A). In the presence of SARS-CoV-2 S proteins, the sandwiched binding was formed by interacting engineered yeast, SARS-CoV-2 S proteins, and reporter AuNPs. The colorimetric signal was obtained by the catalytic reaction of HRP, resulting in the developed whole-cell biosensor that enables the sensitive detection of SARS-CoV-2 S proteins with an LOD of 0.037 μg/mL, approximately 4 × 10^8^ virion particles/mL [177]. 

SPR biosensors have evolved into central tools for characterizing and quantifying biomolecular interactions, and studies leveraging SPR in conjunction with nanobodies have emerged in the research on SARS-CoV-2 POC applications [198]. Researchers have designed an optical biosensor in the portable SPR POC device using a nanobody-modified SPR chip. The objective was to detect SARS-CoV-2 in nasopharyngeal samples. The label-free sensing capability of plasmonic-based sensors enables real-time detection with a response as short as a few minutes [184]. A paper-based POC approach using nanobodies has also been developed for SARS-CoV-2 diagnosis. It was designed as a bifunctional fusion protein with a cellulose-binding domain (CBD) and RBD-specific nanobody (Figure 12B). In this study, Ty1 nanobody was utilized to capture SARS-CoV-2 from complex fluids by immobilizing cellulose materials with functionally oriented nanobodies. As proof of concept, an immunoassay was conducted on cellulose-based filter paper to detect RBD using these bifunctional proteins. The capture efficiency was validated using anti-human Fc-HRP and the 3,3′-Diaminobenzidine Tetrahydrochloride (DAB) substrate with RBD-Fc as a proxy for SARS-CoV-2. Notably, the most intense staining was observed with the combination of nanobody-CBD-coated discs and RBD-containing medium [188].

The unique properties of AuNPs, such as color changes based on the LSPR with distance between particles, are often utilized in detection studies. AuNP-based sensors provide distinctive characteristics that eliminate the need for fluorescent labeling, washing, or enzymatic amplification. The sensors effectively transduce signals related to antigen binding through physical processes, such as AuNP aggregation and sedimentation (Figure 12C). The resulting antigen-dependent optical extinction can be easily detected using spectrometry or portable electronic circuitry. The multivalent nanobody-based AuNP sensors, called nanobody-functionalized nanoparticles for rapid electronic detection (Nano2RED), have been developed to reliably transduce antigen-binding signals upon mixing physical AuNP aggregation and sedimentation. When targeting the SARS-CoV-2 RBD in the S protein, this sensor showed rapid readout within minutes and a high sensitivity with an LOD of 1.3 pM for RBD in diluted human serum [181]. Another example of particle-based methods is nanobodies and magnetic beads used for SARS-CoV-2 detection. In a study, nanobodies were evaluated using MagPlex sandwich fluid array assays, a multiplexed immunoassay on color-coded magnetic microspheres to detect the N protein. Using the selected bivalent pair, one nanobody was immobilized on the microsphere, while the other functioned as the biotinylated recognition reagent. This method yielded an impressive achievement, with an LOD as low as 50 pg/mL for the recombinant N protein [182].

In addition to these innovations, a recent breakthrough has been achieved in developing a nanophotonic biosensor capable of quantitative detection of viral particles in less than 20 min. Based on the bimodal waveguide (BiMW) interferometric technology, this nanosensor has been equipped with novel bioengineered nanobodies targeting the SARS-CoV-2 RBD. The BiMW technology employs a single straight waveguide, generating an evanescent field highly sensitive to minute changes in the refractive index (RI), as observed during a biointeraction event. This method relies on the direct capture of viral particles, and its optimized approach enables exceptional sensitivity in detecting SARS-CoV-2 [186].

#### 3.2.2. Electrochemical Sensing

Sensing approaches employing nanobodies have progressively advanced in accuracy and speed. Furthermore, there is a broader aim to integrate biology into various other disciplines. In pursuit of this integration, nanobodies have been incorporated into the electrochemical field for enhanced sensitivity and rapid signal detection.

**Figure 13:**
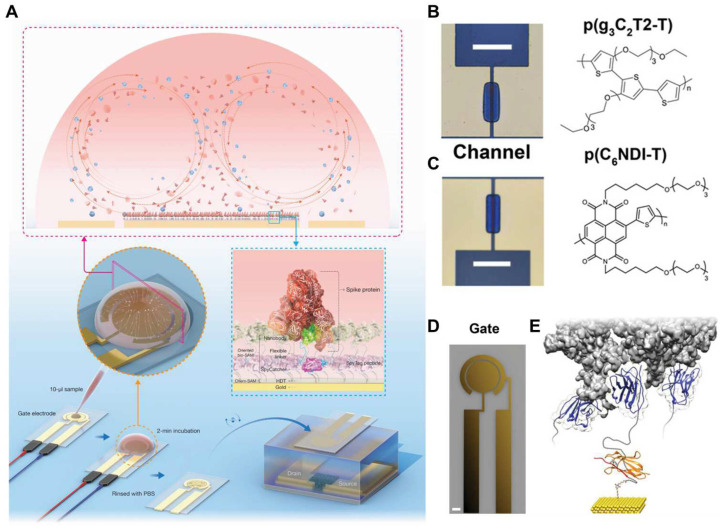
The component and operational principles of the nanobody-organic electrochemical transistors (OECT) biosensor. (**A**) Schematic and operation steps of the alternating current electrothermal flow (ACET) enhanced nanobody-OECT biosensor. The channel was composed of (**B**) p-type p(g3C2T2-T) and (**C**) n-type p(C6NDI-T). The chemical structures of the polymers were depicted alongside the images. (**D**) The circular gate electrode is encircled by a gold layer to facilitate the application of ACET. Scale bar of (**B**–**D**): 100 μm. (**E**) The structural arrangement of the biorecognition unit on the gate electrode. The SpyTag was chemically linked to hexanedithiol monolayer assembled on the gold electrode. The nanobody-SpyCatcher fusion protein binds to this chemical layer through autocatalytic conjugation of a covalent SpyCatcher-SpyTag isopeptide bond. Three Ty1 nanobody structures were depicted in a complex with the trimeric S protein of SARS-CoV-2 (partially shown in grey, based on PDB entry 6ZXN). Reproduced with permission from [190]. Copyright © 2022 Wiley.

First, organic electrochemical transistors (OECT) were combined with nanobodies to detect SARS-CoV-2. In such research, nanobody-functionalized OECT was designed with a modular architecture, enabling the rapid quantification of specific antigens at levels ranging from single-molecule to nanomolar concentrations in complex bodily fluids (Figure 13). The sensor utilizes a conjugated polymer, which can be processed in solution in the transistor channel and high-density, orientation-controlled bioconjugation of nanobody-SpyCatcher fusion proteins on disposable gate electrodes. These devices yield results within 10 min of exposure to 5 μL of unprocessed samples. They maintain high specificity and single-molecule sensitivity in human saliva and serum. Furthermore, these sensors can be reprogrammed to detect any protein antigen, given the availability of a corresponding specific nanobody [195]. The following research focused on overcoming the limitation of conventional biosensors, which depend on the diffusion-dominated transport of the target analyte to the sensor surface. To address this issue, researchers integrated alternating current electrothermal flow (ACET) into an OECT-based sensor to expedite device operation. ACET was applied to the gate electrode functionalized with nanobody-SpyCatcher fusion proteins. With this approach using the SARS-CoV-2 S protein in human saliva, the results showed that ACET facilitated protein recognition within 2 min of sample exposure, affirming its applicability in clinical settings. The ACET-integrated sensor exhibited superior selectivity, higher sensitivity, and lower LOD than the equivalent sensor under diffusion-dominated operation [190].

Second, the nanopore sensor was combined with nanobodies to develop the SARS-CoV-2 diagnostic tool. Nanopores serve as label-free single-molecule analytical tools with significant potential for stochastic sensing of proteins. Direct protein detection is achieved by monitoring the current modulations induced during translocation through the nanopore or binding inside its lumen. In this study, a ClyA nanopore was functionalized with Ty1 nanobody, specifically designed to recognize the SARS-CoV-2 S protein, using a 5–6 nm DNA linker at its periphery. The Ty1-modified nanopores could stochastically sense the S protein. In the proposed model, the motion facilitated by a DNA linker occurs inside the nanopore with unbound nanobodies, resulting in reversible blockade events. However, there is a diminished dynamic movement of nanobodies and an increased current through the open pore upon interaction with S proteins outside the pore. In the present approach, the attached nanobodies on the nanopore act as gatekeepers to prevent the entry of non-cognate proteins, significantly reducing the interference of other components in blood and increasing the sensing selectivity. This nanopore sensor showed the potential for sensitive detection against biomarkers, bacteria, or viruses in clinical samples [192].

Finally, airborne transmission through virus-laden aerosols represents a dominant pathway for the spread of respiratory diseases, including SARS-CoV-2. The direct and non-invasive screening of respiratory virus aerosols in patients posed a persistent technical challenge. To address this issue, researchers have developed a POC testing platform capable of directly detecting SARS-CoV-2 aerosols with just two exhaled breaths from patients, providing results in under 60 s. This innovative platform integrated a handheld breath aerosol collector and a llama-derived nanobody targeting the SARS-CoV-2 S protein. The nanobody was bound to an ultrasensitive micro-immunoelectron (MIE) biosensor designed to detect oxidation of tyrosine amino acids found in SARS-CoV-2 viral particles. The MIE biosensor was connected to a potentiostat, and square-wave voltammetry was employed to oxidize tyrosine and measure the peak oxidation current, indicating the presence of virus aerosols in the sample [193].

#### 3.2.3. DNA Nanotechnology with Nanobodies

Recent developments highlight the diversity and depth of this innovative approach, encompassing not only therapy and detection but also method development in various areas, including mechanical force measurement. In this dynamic landscape, the integration of nanobodies adds another layer of versatility. Despite their small size, nanobodies exhibit enzymatic and pH stability. Coupled with DNA nanostructures, they overcome size limitations, and precise spatial adjustments are achieved by utilizing the DNA nanostructure as a scaffold. As research advances, the combination of DNA nanostructure with aptamers and nanobodies demonstrates infinite possibilities for shaping the future of biotechnology and medical research.

Similar to aptamers, nanobodies serve as excellent bioprobes and find applications as sensors and therapeutic agents. Using DNA nanostructure, spatial adjustments can be made to appropriately position nanobodies on antigens. Despite these advantages, the combination of DNA nanostructure-nanobody for SARS-CoV-2 detection has not been widespread until now. However, the potential power of nanobodies is vast. Considering these characteristics, a viral attachment-blocking chimera (VirABloC) has been developed for SARS-CoV-2, consisting of a nanobody and icosahedral DNA origami termed “snub cube” [199], which provides a large volume, low density, and spatial separation of potential conjugation sites [200].

From an alternative standpoint, substituting the probe with nanobodies, akin to the effective response observed in the context of aptamers when combined with DNA nanostructures for SARS-CoV-2, might yield even more favorable outcomes in specific scenarios. Nanobodies exhibit notable advantages in terms of enzymatic and pH stability, suggesting potential effectiveness in biosensing and bioimaging applications within the physiological environment. However, it is crucial to highlight that modifications are imperative for linking DNA nanostructures and nanobodies. In such situations, employing aptamers capable of facilitating smooth base pairing-based conjugation could provide insights for designing highly effective applications. Developers have the flexibility to craft solutions tailored to specific circumstances by strategically leveraging the strengths and weaknesses of both approaches.

### 3.3. Applications of Nanobodies for SARS-CoV-2 Therapeutics

Amid the global spread of SARS-CoV-2, the scientific community became involved in the search for effective therapeutics to alleviate the adverse effects of viral infection. Utilizing mAbs has emerged as the gold standard for practical treatment approaches [201], representing a class of medicinal compounds that have demonstrated efficacy in treating SARS-CoV-2 infections [202]. Following recent advancements in bioprobe development, nanobodies are emerging as promising candidates to replace antibodies due to several advantages. These include their compact size (approximately 15 kDa), high solubility, stability, ease of adaptability for bioengineering into multivalent configurations, and suitability for cost-effective production using efficient microbial systems [203]. While recombinant Abs are commonly synthesized using mammalian cell culture processes, significant technological effort and comparatively high costs are required for their production [204]. Additionally, although hybridoma technology enables the generation of large quantities of mAbs, it is a time-consuming process (6–9 months) that demands substantial resources and time [205]. In contrast, nanobodies, with their small size and simple structure, offer advantages in terms of expression and purification [206,207], resulting in a more cost-effective production process [159,208]. Notably, nanobodies can be efficiently produced in bacterial expression systems, such as *E. coli*, further reducing production costs [209]. This lower production cost renders nanobodies an attractive alternative for diagnostic and therapeutic applications. Moreover, due to their robust physicochemical characteristics, nanobodies can be delivered via inhalation, making them appealing therapeutic agents for treating respiratory viruses [210,211]. Numerous studies have reported the discovery of nanobodies targeting SARS-CoV-2 for both treatment and diagnosis.

#### 3.3.1. Suppressing Initial Attachment: S Protein S1 Targeting Nanobodies

Various neutralizing antibodies and inhibitors have been recognized for targeting RBD and NTD [212,213,214,215]. The S1 plays a pivotal role in establishing protective immunity against SARS-CoV-2 infection [216]. Dandan et al. discovered the nanobody Nb-H6 through naive phage display, demonstrating broad affinity for both S1 and RBD. Nb-H6 maintained its binding capability across various pH levels and temperatures. Furthermore, it exhibited the ability to neutralize both pseudo-typed and authentic SARS-CoV-2, as well as its variants (Alpha, Delta, Lambda, and Omicron) [216]. Maeda et al. induced immune responses in alpacas to generate a nanobody library. Through phage display, they identified nanobodies binding to the RBD of the S protein. The authors provided a panel of nanobodies for broadly neutralizing five SARS-CoV-2 VOCs. Structural analysis via cryo-electron microscopy (cryo-EM) was performed, particularly for clones p17 and p86, revealing the binding conformation between RBD and nanobody with capped RBD regardless of their up or down conformations [217]. Aksu et al. aimed to develop nanobodies with high affinity for RBD variants in a mutation-tolerant manner. The anti-RBD nanobody Re32D03 discovered by this group demonstrated neutralization against various variants, including Alpha, Beta, Gamma, Epsilon, Kappa, Delta, and Omicron. Additionally, the group utilized an inhalation method to deliver nanobodies directly to the infection site most effectively. The results of administering aerosols to hamsters confirmed their resistance to SARS-CoV-2. This was effective even when applied 24 h after infection. Therefore, inhaled nanobodies not only provide protection against the virus but also alleviate established infections [218]. Ye et al. identified the nanobody Nanosota-1 from a camelid nanobody phage display library. This nanobody also binds to RBD, thereby blocking interaction with hACE2. Nanosota-1C-Fc, with an added Fc tag, exhibited high thermal stability, demonstrated excellent affinity (binding ≈3000 times more tightly to RBD than to ACE2), and had an inhibitory effect on SARS-CoV-2 pseudovirus with a 160-fold improvement. When assessing the therapeutic effects of Nanosota-1C-Fc in hamster and mouse models, it displayed high in vivo stability and tissue bioavailability. Therefore, Nanosota-1C-Fc holds promising therapeutic potential as an alternative for the treatment of COVID-19 [219].

#### 3.3.2. Membrane Fusion Inhibition Strategies: S Protein S2 Targeting Nanobodies

The majority of COVID-19 therapeutic neutralizing antibodies (NAbs) inhibit the SARS-CoV-2 S protein S1 interaction with a human host receptor. However, the development of SARS-CoV-2 immune escape variants, which frequently have mutations in the S protein, could make present S1 NAbs useless. The S protein S2 has a more conserved component, and S2 targeting NAbs provide a wider neutralizing power against different SARS-CoV-2 variations [220]. Wang et al. discovered the nanobody S102 through an alpaca immune library and demonstrated a high affinity at the sub-picomolar level. S102 recognized a conserved epitope in the S2 and was demonstrated to inhibit the cell membrane fusion process through cell fusion assay. As per the mentioned mechanism above, authors speculated that S102 could be bound to the stem helix region close to HR2, blocking the structural change aimed at forming the 6-HB and inhibiting membrane fusion [221].

#### 3.3.3. Multivalent Forms of Nanobodies for the SARS-CoV-2 Therapeutic Applications

Nanobodies represent inherent candidates for anti-SARS-CoV-2 drugs. The functional binding affinity of these natural nanobodies can be enhanced by engineering multivalent nanobodies, allowing them to simultaneously target multiple sites on viral proteins [222]. Huo et al. employed phage display with an immunized llama library to discover four types of nanobodies (C5, H3, C1, and F2) demonstrating affinities in the picomolar range (20–615 pM). Furthermore, they utilized cryo-EM to map these nanobodies onto the RBD. For C5, C1, and H3, they crafted homotrimers using a glycine-serine flexible linker, (GS)_6_, and these trimeric forms could neutralize the Victoria, Alpha, and Beta variants. C5 was engineered to form a C5-Fc fusion by binding with human IgG1 Fc, increasing its half-life. When delivered through the respiratory route in a Syrian hamster model, therapeutic efficacy was confirmed [197]. Xiang et al. utilized proteomics on llama serum immunized with the RBD to generate anti-S protein nanobodies. These nanobodies exhibited affinity levels ranging from picomolar to femtomolar and demonstrated efficacy in inhibiting viral infection at ng/mL levels. Additionally, the researchers engineered multivalent forms (either homo- or heterodimer, or homotrimer) using the discovered nanobodies, employing flexible amino acid linkers. The homotrimeric constructs of Nb213 showed approximately a 30-fold improvement in inhibition efficiency in the pseudovirus luciferase assay. Additionally, a four-fold increase in potency was observed in the heterodimeric constructs (i.e., Nb21-Nb34). The multivalent constructs of nanobodies represent the most potent SARS-CoV-2 neutralizers [203].

Two pan-SARS-CoV-2 and pan-sarbecovirus nanobodies, R14 and S43, were presented by Liu et al. and continued to have neutralizing activity even after aerosolization. Before SARS-CoV-2 infection, administering both nanobodies intraperitoneally dramatically decreased lung viral RNA. The researchers designed various constructs for these nanobodies, such as decameric ones fused to the Fc of IgM (MR14 and MS43), homotrimeric ones made via tandem-repeat constructions (TR14 and TS43), and dimeric derivatives linked to the Fc of IgG1 (DR14 and DS43). Notably, both trimeric and decameric constructs exhibited comparable activity after aerosolization and showed stronger antiviral effects against the Omicron sub-variants. Subsequent structural studies demonstrated how the multivalent derivatives significantly boosted the inhibitory potencies of R14 and S43, revealing their neutralizing processes. Thus, these results indicate that R14, S43, and their multivalent derivatives have great potential as easily inhalable medicines that might successfully target a wide range of SARS-CoV-2 variations and possibly new sarbecovirus isolates [223]. Güttler et al. discovered anti-RBD nanobody monomers capable of inhibiting SARS-CoV-2 infection even at 17 pM (0.2 μg/mL) using alpaca immune libraries. Among 45 nanobodies, some maintained stability even at 95 °C. Additionally, they engineered trimeric forms of nanobody using collagen fragments, which neutralized the virus at a low concentration of 0.6 pM. Moreover, these trimeric nanobodies exhibited superior avidity toward S protein homotrimers compared to bivalent IgG, demonstrated efficient expression in bacteria or yeast, and lacked N-glycosylation sites [224]. Jiang et al. presented a method guided by structural considerations to create a self-assembled trivalent nanobody cluster directed toward the SARS-CoV-2 S protein. They created a tribody, and the monovalent component (Nb6) exhibited high binding affinity (Kd < 1 pM). Moreover, its neutralization capacity against SARS-CoV-2 pseudovirus was 900 times more potent. Cryo-EM confirmed that each monomer of the tribody was bound to the trimeric RBD in the S protein [222].

Several studies demonstrated the utility of nanobodies in the treatment of SARS-CoV-2. The compact size, stability, and ease of engineering make nanobodies advantageous for creating multivalent forms, and they exhibit effective performance against the trimeric S protein.

## 4. Concluding Remarks and Outlook

This comprehensive review explored the transformative landscape of advanced bioprobes, specifically aptamers and nanobodies, and their role in both diagnostic and therapeutic applications for SARS-CoV-2. As we navigate the evolving landscape of MREs, the traditional gold standard (Ab-based platforms) is gradually being replaced by innovative aptamer- and nanobody-based counterparts.

The intrinsic advantages of these advanced MREs, such as high stability, compact size, facile modification, and conformational switching, are extremely useful in a diverse array of sensing platforms. Beyond their utility in diagnostics and therapeutics, these bioprobes facilitate seamless integration into organic bioelectronic materials and devices, promising enhanced rapid performance, sensitivity, and accuracy across various domains.

Our overview of the reported sensing platforms revealed a predominant use of aptamers in diagnostic applications, leveraging their facile modification for sensor development. In contrast, nanobodies, characterized by higher intracellular stability, found well-established roles in therapeutic applications. In terms of size and stability, both aptamers and nanobodies emerged as formidable probes, offering significant potential for further development. 

Of course, some challenges still exist in aptamer- or nanobody-based diagnosis and therapy of COVID-19, particularly concerning bioprobe stability, reproducibility, and sample complexities. Ensuring the stability of aptamers and nanobodies under varying environmental conditions is crucial for reliable performance. Challenges persist in maintaining their structural integrity and functionality. Additionally, achieving consistent performance across different batches of bioprobes and scaling up production to meet the demand for widespread diagnostic testing are significant challenges. Simplifying sample preparation procedures while maintaining detection sensitivity is essential for point-of-care and high-throughput testing applications. Finally, streamlining sample processing workflows without compromising assay performance remains a challenge.

Various strategies, such as chemical modifications, encapsulation techniques, and novel storage conditions, are being explored to improve the stability of aptamers and nanobodies, thereby enhancing their utility in real-time diagnostics. Furthermore, integration with advanced technologies leveraging advancements in microfluidics, nanotechnology, and bioinformatics facilitates the development of aptamers and nanobodies into innovative platforms. Miniaturized biosensing devices enable rapid, sensitive, and multiplexed detection of viral biomarkers in clinical samples. The development of portable, user-friendly diagnostic devices capable of real-time detection holds promise for decentralized testing and remote monitoring of SARS-CoV-2 infections. Aptamer- and nanobody-based biosensors offer the potential for rapid on-site diagnosis in resource-limited settings. Beyond diagnostic purposes, aptamers and nanobodies exhibit therapeutic potential as antiviral agents or targeting moieties for drug delivery systems. Dual function bioprobes capable of both diagnosis and therapy represent an emerging paradigm in combating viral infections.

The collaborative efforts of researchers provided a comprehensive repertoire of MREs, allowing sensor developers to strategically choose from antibodies, aptamers, and nanobodies to meet the specific needs of diverse applications. This review not only highlights the current state of advanced bioprobes but also serves as a guide for future directions, encouraging researchers and developers to leverage the unique attributes of aptamers and nanobodies for continued advancements in the countermeasure against SARS-CoV-2 and beyond.

## Figures and Tables

**Table 1:** Comparison of aptamer, nanobody, and antibody.

	Aptamer	Nanobody	Antibody	Refs.
Composition	ssDNA or RNA	Protein	Protein	[23,24,25]
Affinity (Kd)	nM-pM	nM-pM	nM-pM	[26,27,28]
Specificity	High	High	High	[25,29]
Target type	Cell, virus, bacteria, protein, peptide, small molecule	Cell, virus, bacteria, protein, peptide, small molecule	Cell, virus, bacteria, protein, peptide, small molecule	[24,25,30,31,32]
Molecular weight	Small (≈30 kDa)	Small (12–15 kDa)	Relatively large (≈180 kDa)	[29,33]
Isolation process	In vitro (2–8 weeks)	In vitro or in vivo (2–24 weeks)	In vivo (≈24 weeks)	[25,27,34]
Nuclease degradation	Vulnerable	Resistant	Resistant	[23,25]
Protease degradation	Resistant	More resistant than antibody	Vulnerable	[27,34]
Production cost	Cheap	Cheap	Expensive	[24,25,27]
Modification for application	Convenient	Convenient	Limited	[26,30]
Immunogenicity	Low	Low	High	[25,35]
Stability (pH, temperature)	Stable	Stable	Unstable	[24,33,34]

**Table 2:** The list of aptamer-based assays and biosensors.

Transducer	Sensing Method	Signal Output	Aptamer ID	Sample Type (Binding Site)	Limit of Detection (LOD)	Refs.
Optical	Molecular assay	Fluorescence, FLAA	C7, C9	S protein	41.87 nM	[77]
		Fluorescence, ELISA	Apt 58, Apt 61	N protein	33.28 pg/mL	[78]
		Colorimetric, ELISA	MSA1	Pseudovirus (S protein S1)	400 fM	[79]
		Chemiluminescence, ELISA	SNAP 1, SNAP1.50	UV-inactivated virus (S protein NTD)	5 × 10^5^ copies/mL	[80]
	Nanoparticle-based assay	Fluorescence, bead-based sandwich-type assay	SpS1-C1, SpS1-C4	Pseudovirus (S protein S1)	3.9 × 10^3^ pseudoviral particles/mL	[81]
		Fluorescence, GNS-NP-SET	CoV2-RBD-4C	Pseudovirus (S protein RBD)	130 fg/mL (RBD), 8 particles/mL (virus)	[82]
		Colorimetric, bead-based sandwich-type assay	SpS1-C1, SpS1-C4	Pseudovirus (S protein S1)	1 × 10^1^ pseudoviral particles/mL	[81]
		Colorimetric, AFGN	CoV-2-RBD-4C	Inactivated virus (S protein)	16 nM (S protein), 3540 genome copies/μL (inactivated virus)	[83]
		LSPR	S1 aptamer, S1 aptamer-T, and N aptamer-T	S protein S1	0.26 nM	[84]
		LSPR	T-shaped aptamer	N protein	9.2 pM	[85]
		SERS, Nanopopcorn substrate	aptamer DNA	S protein	10 PFU/mL	[86]
		SERS, Silver nanoforest substrate	SpS1-C1, SpS1-C4	WT, Delta, and Omicron S protein S1	240 fM, 240 aM	[73]
		SERS, Nanoparticle substrate	CoV2-RBD-1C, CoV2-RBD-4C, CoV2-6C3, rank 8	Pseudovirus (S protein RBD)	0.7 fg/mL (RBD), 0.8 TU/mL (pseudovirus)	[87]
	Biosensor	Colorimetric, RCA-ULS	TMSA52	Pseudovirus (Omicron S protein)	3.2 × 10^3^ copies/mL	[88]
		Fluorescence, MES	Aptamer1, SNAP1	Pseudovirus (S protein)	37 active virions/μL	[89]
		nanoFPI	WTapta1, BEapta1, KAapta1	Pseudovirus (S protein RBD)	4 TCID_50_/mL	[90]
		BLI	tNSP3	N protein	4.5 nM	[91]
		SPR	apt-1C	S protein	36.7 nM	[92]
		SPR	Apt 58	N protein	4.9 pg/mL	[93]
	Point-of-care (POC)	Fluorescence, fluorimeter	CoV2-RBD-1C, CoV2-RBD-4C	Pseudovirus (WT S protein-RBD)	1 × 10^6^ copies/mL (D614G), 1 × 10^7^ copies/mL (B.1.1.7)	[94]
		Colorimetric	-	SARS-CoV-2 Alpha, Beta, Delta, and Gamma variant	100 ng/mL	[95]
		Colorimetric, aptamer-hybrid ELISA	Np-A48, Np-A58	N protein	20 pM	[96]
		Colorimetric, LFA	SNAP1, SNAP4.74	UV-inactivated virus (S protein NTD)	1 × 10^6^ copies/mL	[97]
		Colorimetric, LFA	SNAP4.74, SCORe.50	Wild-type S protein (S protein NTD), Omicron S protein (RBD)	100 pM (WT S protein), 50 pM (Omicron S protein)	[98]
		Colorimetric, ELAAA and hybrid-LFS	Apt #6	N protein	0.1 ng/mL and 0.1–0.5 ng/mL	[99]
	Other	Fluorescence, ACNAA	Aptamer O1, O2	Pseudovirus S protein	7.5 × 10^3^ PFU/mL	[100]
		Fluorescence, FQc	CoV2-RBD-1C, CoV2-RBD-4C	S protein and RBD	-	[101]
Electro-chemical	Biosensor	SWV, screen-printed GE	CoV2-RBD-1C	S protein S1	0.75 fM	[102]
		SWV, screen-printed GE	CoV2-RBD-1C, CoV2-RBD-4C	S protein and RBD	-	[101]
		DPV, 3 mm diameter GE	Apt1, Apt2	S protein	9.79 fg/mL	[103]
		DPV, FMEA chip	C7, C9	S protein	8.85 fg/mL (0.07 fM)	[104]
		DPV, QDs-Apta-MB electrochemical probe	nCoV-S1-A1	S protein RBD	0.5 pg/mL	[105]
		DPV, SWCNTs electrode	CoV2-6C3	S protein RBD	7 nM	[106]
		EIS, AuNP-CE	CoV2-RBD-1C	S protein RBD	1.30 pM (66 pg/mL)	[107]
		EIS, Au@UiO-66-NH_2_ on GE	Np-A48t, Np-A61t	N protein	0.31 pg/mL	[108]
		Two-terminal I–V characteristics, CNT-array TFT	CoV2-RBD-1C, CoV2-RBD-4C, CoV2-6C3	Wild-type S protein RBD, Omicron S protein RBD	10 aM (WT S protein), 6 aM (Omicron S protein)	[109]
		ICR, AFNCs	XN-268s	S protein S1	1 fM	[110]
	Point-of-care (POC)	Glucometer	N aptamer 1, CoV2-RBD-1C	N protein, S protein	In buffer 1.50 pM (N protein), 1.31 pM (S protein) and in saliva 5.27 pM (N protein), 6.31 pM (S protein)	[111]
		EIS, Mobile-operated potentiostat	Dimerization with MSA1T and MSA5T	Pseudovirus (S protein S1)	1 fM (WHTS), 2.8 fM (UKTS), 3.6 fM (INTS), 1 × 10^3^ copies/mL (WHPV), 5 × 10^3^ copies/mL (UKPV)	[112]

Abbreviation: FLAA, fluorescence-linked aptamer assay; ELISA, enzyme-linked immunosorbent assay; GNS-NP-SET, gold nanostar-based distance-dependent nanoparticle surface energy transfer; AFGN, aptamer-functionalized gold nanoparticle; LSPR, localized SPR; SERS, surface-enhanced Raman scattering; RCA-ULS, rolling circle amplification-urease-based litmus sensor; MES, Microfluidic Enrichment Strategy by stoichiometric balanced DNA computation; NanoFPI, nanostructured Fabry–Perot interference; TCID_50_, 50% tissue culture infectious dose; BLI, BioLayer Interferometry; SPR, surface plasmon resonance; LFA, lateral flow assay; ELAAA, Enzyme-Linked Aptamer–Antibody sandwich Assay; hybrid-LFS, hybrid lateral flow strip; ACNAA, Aptamer-Capped Nanoporous Anodic Alumina; FQc, Fluorophore–Quencher complex; DPV, Differential Pulse Voltammetry; SWV, square-wave voltammetry; ICR, Ionic Current Rectification; EIS, Electrochemical Impedance Spectroscopy; FMEA chip, Flexible Multi-Electrode Arrays chip; GE, gold electrode; AFNCs, Aptamer-Functionalized NanoChannels; AuNP-CE, Gold NanoParticle-coated Carbon Electrode; SWCNTs electrode, Single-Walled Carbon NanoTube electrodes; QDs-Apta-MB, Quantum Dots-Aptamer-Molecular Beacon; CNT-array TFT, carbon-nanotube-array-based thin-film transistor; Au@UiO-66-NH_2_, gold nanoparticle [AuNP]-decorated amino-functionalized UiO-66; WHTS, WuHan variant (wild-type) Trimeric Spike protein; UKTS, UK variant Trimeric Spike protein; INTS, INdian variant Trimeric Spike protein; WHPV, WuHan variant (wild-type) pseudo-typed virus; UKPV, UK variant pseudo-typed virus.

**Table 3:** The list of nanobody-based assays and biosensors.

Transducer		Signal Output	Nanobody ID	Sample Type (Binding Site)	Limit of Detection (LOD)	Refs.
Optical	Molecular assay	Yeast-based ELISA	Nb33	S protein S2	0.037 μg/mL (about 4 × 10^8^ virion particles/mL)	[177,178]
		ELISA	C5, F2	Pseudovirus (RBD)	16 TCID50/ml	[176,179]
		Sandwich ELISA	NTD E4-3, NTD B6-1	N protein	4 ng/mL	[180]
	Nanoparticle-based assay	Nano2RED	RBD8, RBD10	RBD	1.3 pM (~40 pg/mL)	[181]
		MagPlex fluid array assays	E2–C2, E2–E2	Killed Virus (N protein)	50 pg/mL (1.28 × 10^3^ PFU/mL)	[182]
	Biosensor	LSPR	NB1D6, NB4E9	RBD	0.01 ng/mL	[183]
		SPR	VHH 72	Viral Particles (RBD)	2.9 × 10^4^ viral particles/mL	[184,185]
		BiMW	1.26	Pseudovirus (RBD)	178 TCID50/mL	[186,187]
Electro-chemical	Biosensor	PBD	Ty1	Pseudovirus (RBD)	Unknown	[188,189]
		ACET	Ty1	RBD	1 fM at V_G_ = 0.5 V	[189,190]
		PLA	Ty1	S protein (RBD)	200 pM	[189,191]
		Nanopore sensing	Ty1	S protein (RBD)	115 pM	[189,192]
		MIE biosensor	NIH-CoVnb-112	Inactivated Virus (RBD)	6–32 RNA copies/mL	[193,194]
		OECT	VHH 72	RBD, S protein S1	48 fM (RBD), 18 zM (S protein S1)	[185,195]
		PEC immunosensor	Nb11-59	S protein (RBD)	5 fg/mL	[196,197]

Abbreviation: Nano2RED, nanobody-conjugated nanoparticles for rapid electronic detection; BiMW, bimodal waveguide; PBD, paper-based diagnostics; ACET, alternating current electrothermal flow; PLA, proximity ligation assay; MIE biosensor, micro-immunoelectrode biosensor; OECT, organic electrochemical transistors; PEC immunosensor, photoelectrochemical immunosensor.

## Data Availability

Data are contained within the article.
